# Time-Frequency Feature Representation Using Multi-Resolution Texture Analysis and Acoustic Activity Detector for Real-Life Speech Emotion Recognition

**DOI:** 10.3390/s150101458

**Published:** 2015-01-14

**Authors:** Kun-Ching Wang

**Affiliations:** Department of Information Technology & Communication, Shih Chien University, 200 University Road, Neimen, Kaohsiung 84550, Taiwan; E-Mail: kunching@mail.kh.usc.edu.tw; Tel.: +886-76-678-888-5723; Fax: +886-76-678-888-4332

**Keywords:** multi-resolution, discrete wavelet transform, time-frequency texture, acoustic activity detection, spectrogram, Laws masks

## Abstract

The classification of emotional speech is mostly considered in speech-related research on human-computer interaction (HCI). In this paper, the purpose is to present a novel feature extraction based on multi-resolutions texture image information (MRTII). The MRTII feature set is derived from multi-resolution texture analysis for characterization and classification of different emotions in a speech signal. The motivation is that we have to consider emotions have different intensity values in different frequency bands. In terms of human visual perceptual, the texture property on multi-resolution of emotional speech spectrogram should be a good feature set for emotion classification in speech. Furthermore, the multi-resolution analysis on texture can give a clearer discrimination between each emotion than uniform-resolution analysis on texture. In order to provide high accuracy of emotional discrimination especially in real-life, an acoustic activity detection (AAD) algorithm must be applied into the MRTII-based feature extraction. Considering the presence of many blended emotions in real life, in this paper make use of two corpora of naturally-occurring dialogs recorded in real-life call centers. Compared with the traditional Mel-scale Frequency Cepstral Coefficients (MFCC) and the state-of-the-art features, the MRTII features also can improve the correct classification rates of proposed systems among different language databases. Experimental results show that the proposed MRTII-based feature information inspired by human visual perception of the spectrogram image can provide significant classification for real-life emotional recognition in speech.

## 1. Introduction

Speech emotion recognition (SER) is one of the most fundamental components for human machine/computer interaction (HCI). SER can be defined as the extraction of the emotional state of the speaker from his or her speech signal. With the exponential growth in available computer power and significant progress in speech technologies, SER has been successfully applied in several HCI domains. Among the HCI, the interface with robots [1–3], call center environments [4] and the entertainment industries have been several potential applications. Many different SER systems have been proposed for the emotion extraction from the speech. These different systems were using the different features and classifiers. It is well-known that two parts—feature extraction and emotion machine classification—are the major computational tasks for the SER system.

In terms of extraction, they have to carry sufficient information about the emotional states of a speaker. So far, a variety of acoustic features have also been explored. In [5], the authors selected the twenty pitches and energies related features to recognize seven emotions in German and English (angry, disgust, fear, surprise, joy, neutral and sadness). In [6], pitch, log energy, formant, band energies and MFCCs were used as base features in a SONY AIBO database. In [7], the authors used pitch, formant, intensity, speech rate and energy related features to classify neutral, angry, laugh and surprise for a 40-sentence corpus. In [8], energy, pitch, zero crossing, phonetic rate, LPCCs and their derivatives, were tested and combined with MFCCs for performing speaker-dependent emotion recognition. In [9], the short time log frequency power coefficients (LFPC) along with MFCCs were adopted as emotion speech features to recognize six emotions in a 60-utterance corpus. In [10], fundamental frequency, energy and audible duration features were extracted to recognize sadness, boredom, happiness and anger in a corpus recorded by eight professional actors. The overall accuracy was only about 50%, but anger and other basic emotions can be successfully discriminated by these features. In [11], the prosodic features derived from pitch, loudness, duration and quality features were extracted to recognize five emotions (anger, happiness, neutral, sadness and bored) in a 400-utterance database. According the above statement, we find that the spectral features and prosodic features are some of popular features and can be used for speech emotion recognition because both of these features contain the emotional information. For example, fundamental frequency, loudness, pitch and speech intensity and glottal parameters are the prosodic features used to model the different emotions [5–7]. Linear predictive cepstral coefficients (LPCC) [8,12] and Mel-frequency cepstral coefficients (MFCC) are some of the spectral features [6,8,9,13]. In [14,15], a 2-D Gabor filter bank was applied to Mel-spectrograms. The author tries to use 2-D spectrogram image instead of 1-D information. The resulting outputs of the Gabor filters were concatenated into two-dimensional vectors and used as features in speech recognition experiments. In [16], a similar method was applied in speech discrimination and enhancement. In recent studies [17–19], a 2-D Gabor filter bank was used to represent speech harmonistics, formants, vertical onsets/offsets, noise and overlapping simultaneous speakers by decomposing localized patches of spectrograms. In fact, the texture-like time-frequency representation derived from 2-D narrowband speech spectrogram usually contains distinctive patterns that capture different characteristics of speech emotion signals. It is a well-known graphical display of the squared magnitude of the time-varying spectral characteristics of speech [20]. The compact and highly efficient representation carries much information about parameters such as energy, pitch F0, formants and timing. These parameters are the acoustic features of speech most often used in emotion recognition systems [21,22]. In 1980 [23], Kenneth Ivan Laws brought forward the Laws' masks idea to compute the texture properties of images. In [24], multi-resolution analysis of discrete wavelet transform has provided to be an effective approach to analyze texture image. In order to obtain the desired frequency band, Chang et al. proposed a multi-resolution approach based on a modified wavelet transform called the tree-structured wavelet transform (TSWT) for texture analysis and classification [25].

In real-life condition, the level of background signal change rapidly. The amplitude of emotional speech signal also varies with the emotional state. In order to increase the accuracy in real-life emotional recognition, a novel feature extraction based on multi-resolution texture image information (MRTII) has been proposed in this paper. First, the strategy of BS-Entropy-based acoustic activity detection (AAD) for detecting voice-activity segments is required in order to extract the correct emotional state especially in real-life condition. We find that the calculation of the spectral entropy parameter implies that the spectral entropy depends only on the variation of the spectral energy but not on the amount of spectral energy. In real-life environment, the spectral entropy parameter is robust against changing signal levels, even though signal amplitude varies with the emotional state. So, the utilized BS-Entropy-based AAD is benefit for real-life emotional recognition in speech. Next, the input speech is decomposed into 24 critical subbnads using five-level 1-D wavelet decomposition. Through the calculation of gray-scale 2-D spectrogram image, the 1-D voice-activity segment is transformed into a recognizable 2-D spectrogram image. Next, the cubic curve is used to enhance the contrast of emotional speech spectrogram images. In order to provide the discrimination between each emotion, the multi-resolution sub-band analysis of tree-structured wavelet transform (TSWT) is then utilized. With the transform, we are able to zoom into any desired frequency channel of each emotion for further decomposition, so the desired sub-band images will contain rich texture information while the emotional speech spectrogram image with TSWT is decomposed into four sub-band images. Consequently, the MRTII feature set can be determined by using Laws' masks on the desired sub-band image for extracting the multi-solution of texture image information.

This paper is organized as follows; in Section 2, we introduce the proposed MRTII-based feature extraction approach for emotion classification in speech. The BS-Entropy-based AAD and the tree-structured wavelet transform (TSWT) are then presented in detail. In addition, the MRTII features using the Laws masks derived from the desired frequency channels are schematically described. Section 3 introduces the emotion database and the existing features such as MFCC, prosodic feature and LLD feature. The database includes short sentences covering the five types of emotions, namely Anger, Sadness, Fear, Neutral and Happiness. The experiments and results are presented in Section 4. Finally, Section 5 provides the discussion and conclusions.

## 2. The Proposed MRTII-Based Features

**Figure 1.**
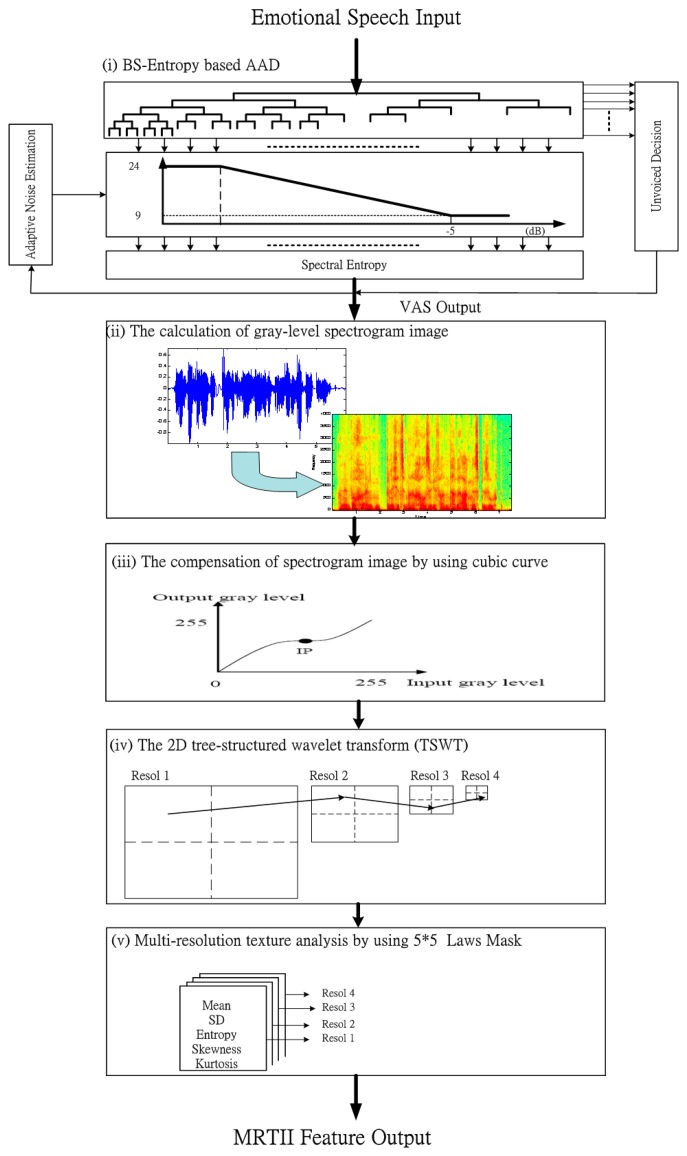
The flowchart for deriving the proposed MRTII-based feature extraction approach.

Figure 1 shows a diagram of the proposed MRTII-based feature extraction algorithm including BS-entropy based AAD and the multi-resolution texture analysis. In the step 1 of Figure 1, the emotional speech is inputted into BS-entropy based AAD. We can find that the voice-activity segment (VAS) is outputted. In step 2, the calculation of gray-level spectrogram image is determined. In step 3: we can compensate the spectrogram image by using cubic curve. Next, through the 2D tree-structured wavelet transform (TSWT), we can complete the multi-resolution analysis for the desired frequency channel. Finally, the multi-resolution texture analysis by using 5 × 5 Laws mask will be done. The details will be addressed in the following subsections.

### 2.1. BS-Entropy Based Acoustic Activity Detection (AAD)

First, the speech signal is high-pass filtered to emphasize the important higher frequency elements. The pre-emphasization is usually done by a high-pass filter. The main use of this process is to flatten the speech signal and to make it less susceptible to finite precision effects later in the signal processing. Commonly, the pre-emphasizer is represented by a first order FIR filter [26]. Next, the speech frame, *x*[*n*] is divided into several segments. The chosen frame size is 256 samples and 50% overlap with neighboring frames. After frame partitioning, the Hamming window is applied to each segment. The purpose of the Hamming window is to minimize the signal discontinuities at the beginning and end of each segment. The Hamming window function is given by [27]:

(1) $$w[n]=\{\begin{array}{ll} 0.54-0.46\cos\left(2n\pi /\left(N-1\right)\right), & \text{0}\leq n\leq N-1\\ 0, & \text{otherwise} \end{array}$$

where *N* is the length of the window. *w*[*n*] is Hamming window.

Each windowed speech segment is then converted into the parametric representations for further analysis. In human computer interface processing, it is important for the system to be able to detect the accurate activity of emotional utterances. The purpose of acoustic activity detection (AAD) is to find the start and the end of voice-activity segments (VAS). In this subsection, our previous work [28] is used to apply into the proposed MRTII-based feature extraction. Through five-layer Bark-scale wavelet decomposition, the 24 critical subbands, widely used in perceptual auditory modeling [29,30], can be determined. Consequently, the spectral energy of the *ξ*th subband on the *m*th frame is evaluated by the sum of squares:

(2) $$E(\xi ,m)=\sum_{\omega_{\xi ,l}}^{\omega_{\xi ,h}}{\left|X(\omega ,m)\right|}^{2}$$

where *X* (*ω*, *m*) means the *ω*th wavelet coefficient. *ω_ξ,l_* and *ω_ξ,h_* denote the lower boundaries and the upper boundaries of the *ξ*th subband, respectively.

According to Wu *et al.* [31], the estimated pure speech signal is a good indicator for detecting voice-activity segment (VAS). The *ξ*th frequency subbands energy of pure speech signal of the *m*th frame *Ẽ*(*ξ*, *m*) is estimated:

(3) $$\tilde{E}(\xi ,m)=E(\xi ,m)-\tilde{N}(\xi ,m)$$

where *Ñ*(*ξ*, *m*) is the noise power of the *ξ*th frequency subband.

It is found that the more the frequency subband is covered by noise the smaller the *Ẽ*(*ξ*, *m*). Since the frequency subband with higher *Ẽ*(*ξ*, *m*) contains more pure speech information, we should sort the frequency subbands according to their *Ẽ*(*ξ*, *m*) value. That is:

(4) $$\tilde{E}(I_{1},m)\geq \tilde{E}(I_{2},m)\geq \cdots \geq \tilde{E}(I_{N_{ub}},m)$$

where *I_i_* is the index of the frequency subband with the *i*th max energy.

The first *N_um_* frequency subbands *I*_1_, *I*_2_,…, *I_N_um__* are selected and denoted as the useful number of frequency subband *N_ub_*, for the succeeding calculation of spectral entropy. According to the relation between the number of useful frequency subbands *N_ub_*(*m*) and *SNR*(*m*), we can see that the number of useful frequency subbands increases with the increase of *SNR*. The relationship between *N_ub_*(*m*) and *SNR*(*m*) can be simulated by a linear function:

(5) $$N_{ub}(m)=\{\begin{array}{cc} 9 & ,\text{SNR}(m)<-5dB\\ \left[(24-9)\times (\left(\text{SNR}(m)-(-5)\right)/(30-(-5)))+9\right] & ,-5dB\leq \text{SNR}(m)\leq 30dB\\ 24 & ,\text{SNR}(m)<30dB \end{array}$$

where [·] is the round off operator and *SNR*(*m*) denotes a frame-based posterior SNR for the *m*th frame.

In addition, *SNR*(*m*) is depended on the all summation of subbnad-based posterior SNR *snr*(*ξ*, *m*) on the *ξ*th useful subband and defined as:

(6) $$\begin{array}{ll} \text{SNR}(m) & =10\log_{10}\sum_{\xi \in N_{ub}}\text{snr}(\xi ,m),\\ \text{where} & \text{snr}(\xi ,m)=E{\left(\xi ,m\right)}^{2}/\tilde{N}(\xi ,m) \end{array}$$

The spectral power of subband noise can be estimated by averaging past spectral power value using a time-frequency dependent smoothing parameter in order to recursively estimate the noise power spectrum, as follows:

(7) $$\tilde{N}(\xi ,m)=\alpha (\xi ,m)\cdot \tilde{N}(\xi ,m-1)+(1-\alpha (\xi ,m))\cdot E(\xi ,m)$$

where *α*(*ξ*, *m*) means the smoothing parameter and be defined as:

(8) $$\alpha \left(\xi ,m\right)=\{\begin{array}{ll} 1, & \text{if}\;\text{VAS}\left(\text{m}-1\right)=1\\ 1/1+e^{-k\cdot \left(\text{snr}\left(\mathrm{\xi i;},m\right)-T\right)}, & \text{otherwise} \end{array}$$

where *_T_* is used for center-offset of the sigmoid transition curve.

The smoothing parameter is set to one when in a previous speech-dominated frame, the spectral power of the subband noise remains a noise-dominated frame. Otherwise, the smoothing parameter may be chosen as a sigmoid function when it is a noise-dominated frame. In addition, the unvoiced segments are also determined as:

(9) $$\begin{array}{ll} S_{\text{unvoiced}} & =\{\begin{array}{ll} 1 & ,\text{if}\;E_{L2}>E_{L1}>E_{L0}\;\text{and}\;E_{L0}/E_{L2}<0.99\\ 0 & ,\text{otherwise} \end{array}\\ \text{where} & \begin{array}{lll} E_{L0}=\sum_{j=1}^{8}W_{j}^{5}, & E_{L1}=\sum_{j=9}^{12}W_{j}^{4}, & E_{L2}=\sum_{j=13}^{18}W_{j}^{4}+W_{19}^{3} \end{array} \end{array}$$

Next, in order to calculate a measure of entropy defined on the spectrum domain of the selected frequency subbands, the probability associated with subband energy is described as follows:

(10) $$P(\xi ,m)=E(\xi ,m)/\sum_{\omega =1}^{N_{ub}(m)}E(\omega ,m)$$

where *N_ub_*(*m*) is the number of useful frequency subbands.

Applying the above constraints, the spectral entropy *H* (*m*) of frame *m* can be defined as below:

(11) $$H(m)=-\sum_{\xi =1}^{N_{ub}(m)}P(\xi ,m)\cdot \log[P(\xi ,m)]$$

Consequently, the voice activity segment (VAS) is determined in spite of change in amplitude of emotional speech input or in background noise-level.

(12) $$\text{VAS}=\left\{H=1\right\}\cup \left\{S_{\text{unvoiced}}=1\right\}$$

### 2.2. The Calculation of Gray-Level Spectrogram Image

We first present the calculation of gray-level spectrogram image [32], the spectrogram images with time-frequency-intensity representation is generated as below:

(13) $$X(k,t)=\sum_{n=0}^{N-1}\begin{array}{cc} x[n]w[n-t]e^{-2\pi \text{ikn}/N}, & k=0,\ldots \ldots ,N-1 \end{array}$$

where *X* (*k*, *t*) is denoted as time-frequency-intensity representation. *N* is the length of the window.

The log-spectrogram is required owing to the logarithmic nature of the human perception of sound. So, the gray-scale spectrogram is obtained by the log-spectrogram normalized into a grayscale normalized image, within the range from 1 to 255.

(14) $$S_{\log}(k,t)=\log(\left|X(k,t)\right|)$$

(15) RSpIm(k,t)=(Slog(k,t)−Smin)/(Smax−Smin)

where *S*_log_(*k*, *t*) is denoted as log-spectrogram. *R_Sp_*_Im_ (*k*, *t*) is denoted as the spectrogram image representation.

### 2.3. The Compensation of Spectrogram Image

Next, we proposed a procedure to transfer 1D speech signal into 2D gray-level spectrogram image. After the calculation of gray-level spectrogram image, we could compensate the backlight image [33]. We utilized an image compensation curve, which the compensation curve can be achieved with cubic curve equation. The domain and co-domain of this curve was between 0 and 255, respectively.

Based on the assuming that curve must pass through (0, 0) and (255, 255) two points, the compensation curve was set as:

(16) $$y=f(x)=ax^{3}+bx^{2}+cx+d$$

where *x* is the pixel value in the original image, and *y* is the pixel value of the image after adjusting the curve.

The curve was simplified as follows

(17) $$\begin{array}{lll} y=f(x)=ax^{3}+bx^{2}+cx, & \text{while} & f(0)=d \end{array}$$

(18) $$\begin{array}{lll} c=1-a\times 255^{2}-b\times 255, & \text{while} & f(255)=a\times 255^{2}+b\times 255+c \end{array}$$

In Equation (17), the c value was calculated as Equation (18). Thus, we obtained the following equation:

(19) $$y=f(x)=ax^{3}+bx^{2}+\left(1-a\times 255^{2}-b\times 255\right)\times x$$

In order to allow the contrast of the cubic curve, which is the cubic curve function having a horizontal line, the first deviation needs a zero value shown as below:

(20) $$f^{\prime }(x)=0=3ax^{2}+2bx+c$$

The *b*^2^ − 4*ac* = 0 could satisfy this real root domain according to that the characteristics of this quadratic equation *f*′ (*x*) = 0 shows the quadratic equation had a real root. Hence, the Equation (21) is given as follows:

(21) $$b^{2}=3\times a-255^{2}\times 3a^{2}-255\times 3\times a\times b$$

The inflection point (A, B) was set ( $\overset{-b}{\;}/\underset{3a}{\;},f(\overset{-b}{\;}/\underset{3a}{\;})$) while 0 ≤ *A* ≤ 255 and 0 ≤ *B* ≤ 255. So, the following equation is obtained as below:

(22) $$a=\frac{1}{255^{2}-3\times 255\times A+3\times A^{2}}$$

### 2.4. The 2D Tree-Structured Wavelet Transform (TSWT)

**Figure 2.**
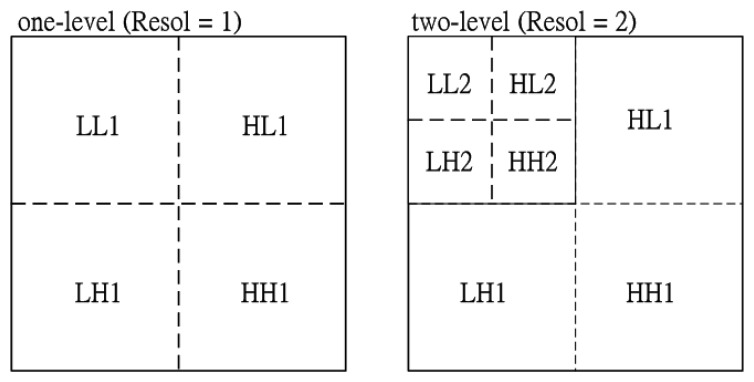
Spectrogram image decomposition: (**a**) one-level; (**b**) two-level.

In this section, multi-resolution texture analysis plays an important role in the 2-D spectrogram image for speech emotional classification. Therefore, a 2-D discrete wavelet transform (DWT) is utilized for spectrogram image decomposition. The image is actually decomposed into four sub-bands and critically sub-sampled by applying DWT as shown in Figure 2a.

These subbands labeled LH1, HL1 and HH1 represent the finest scale wavelet coefficients (regarded as detail images) while the sub-band LL1 corresponds to coarse level coefficients (regarded as the approximation image). To obtain the next coarse level of wavelet coefficients, the sub-band LL1 alone is further decomposed and critically sampled. This results in a two-level wavelet decomposition as shown in Figure 2b. Similarly, to obtain further decomposition, LL2 will be used. This process continues until some final scale is reached. The values or transformed coefficients in approximation and detail images (sub-band images) are the essential emotional features, which are shown here as useful for multi-resolution texture analysis of emotional discrimination.

**Figure 3.**
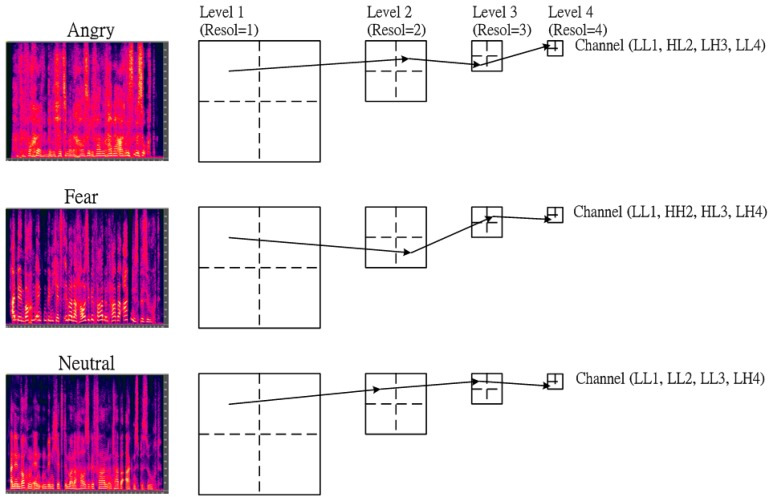
The first prefer channel in 4-level tree-structured wavelet transform domain for three types of emotion: Anger, Fear and Neutral.

In fact, the texture images of various types of emotion are concentrated in different frequency bands. To characterize the discrimination between each emotion, the texture information focused on specific frequency channel must be adaptively depicted. In this subsection, a multi-resolution approach based on a modified wavelet transform or called as tree-structured wavelet transform (TSWT) is then adopted into the proposed SER to obtain the desired frequency channel for further decomposition. The details of the tree-structured wavelet transforms algorithms were given in [25]. In addition, the energy distribution of texture for each emotion is different. For example, Figure 3 shows that the main channel in 4-level tree-structured wavelet transform domain for three types of emotion: Anger, Fear and Neutral. In order to further extend the discrimination between emotions, the first four dominant channels required are shown in Table 1. The dominant frequency channels were summed up across all emotional databases so as to have generic distributions.

We can see from Table 1 that the first two energy distributions in the first two dominant channels are LL1, HL2 for Fear, Neutral and Sadness. Similarly, the energy distribution is LL1, HL2 for Anger and Happiness. In addition, we can find that the energy distribution is almost similar for three emotions: Fear, Neutral and Sadness. In contrast, the channel distribution between Anger and Happiness is also almost similar.

### 2.5. Multi-Resolution Texture Analysis

For the desired frequency channels containing rich texture image, the Laws' texture energy measures (TEM) were used to extract texture property of each channel. The two 2-dimensioanl convolution kernels, generated from different combinations of the 5 masks: h1 = [1, 4, 6, 4, 1], h2 = [−1, −2, 0, 4, 1], h3 = [−1, 0, 2, 0, −1], h4 = [−1, 2, 0, −2, 1], and h5 = [1, −4, 6, −4, 1], are applied onto the converted gray scale spectrogram image. We apply 5×5 dimensional Laws' Mask to produce a total of 25 masks. Through five statistical descriptors of mean, standard deviation (SD), entropy, skewness and kurtosis, we have conducted the statistical evaluation to examine the discrimination between each emotional speech signal using Laws' masks technique.

The five statistical descriptors of mean, standard deviation (SD), entropy, skewness and kurtosis were computed as below:

(23) $$\text{Mean}=\frac{\sum_{i=0}^{M}\sum_{j=0}^{N}\left[TR_{ij}\right]}{M\times N}$$

(24) $$SD=\sqrt{\frac{\sum_{i=0}^{M}\sum_{j=0}^{N}{\left(TR_{ij}-\text{Mean}\right)}^{2}}{M\times N}}$$

(25) $$\text{Entropy}=\frac{\sum_{i=0}^{M}\sum_{j=0}^{N}{\left(TR_{ij}\right)}^{2}}{M\times N}$$

(26) $$\text{Skewnes}s=\frac{\sum_{i=0}^{M}\sum_{j=0}^{N}{\left(TR_{ij}-\text{Mean}\right)}^{3}}{M\times N\times SD^{3}}$$

(27) $$\text{Kurtosis}=\frac{\sum_{i=0}^{M}\sum_{j=0}^{N}{\left(TR_{ij}-\text{Mean}\right)}^{4}}{M\times N\times SD^{4}}-3$$

These five features are used to judge the variation of texture information. Equations (23)–(27) are the calculation formula of the five feature values, where *TR_ij_* represents the unchangeable values within 25 masks from TEM before and after rotation from a spectrogram image *I*_(_*_i_*_,_*_j_*_)_ of size (*M* × *N*). Finally, each equation will have 14-dimensional feature vectors, so a total of five feature vectors are 70-dimensional. According the Table 1, four-levels are required to identify the discrimination between emotions. Therefore, the total feature set, *x*, of four-levels is 280-dimentional while each level is 70-dimensional in feature vectors:

(28) $$\begin{array}{ll} x=\left\{x_{1},x_{2},x_{3},x_{4}\right\} & \\ \text{where}\;x_{i}=\left({\text{Mean}}_{i},SD_{i},\text{Entrop}y_{i},\text{Skewnes}s_{i},\text{Kurtosi}s_{i}\right), & 1\leq i\leq 4 \end{array}$$

Then, the four-level feature vectors will be used as the input for training the emotional classifier.

## 3. Emotion Speech Database and the Existing Feature Extraction

To demonstrate the effectiveness of the proposed MRTII-based feature extraction applied to a SER system, we carried out experiments on the artificial database and real-life database. In the following, our experimental results will be presented.

### 3.1. Artificial Database

#### 3.1.1. EMO-DB

The Berlin Speech Emotion Database (EMO-DB) [34] was recorded at the Technical University, Berlin. It contains seven classes of basic emotions (Anger, Fear, Happiness, Disgust, Boredom, Sadness, and Neutral). Ten professional German actors (five men and five women) spoke ten sentences in German language.

#### 3.1.2. eNTERFACE Corpus

The eNTERFACE corpus is a further public, audio-visual emotion database. It consists of six emotion categories: Anger, Disgust, Fear, Happiness, Sadness, and Surprise [35]. The 42 subjects (eight women) from 14 nations were recorded in English in an office environment.

#### 3.1.3. KHUSC-EmoDB

The recording of the corpus of KHUSC-EmoDB comprises Mandarin language. It is a self-recorded database. Its members are all students from Shih-Chien University. The emotional voices of this corpus are recorded from four women and 13 men. Each speaker is recorded in all four emotions (Happiness, Fear, Sadness and Anger), which are same as the overlap between EMO-DB and eNTERFACE.

### 3.2. Real-Life Database

This subsection showed the evaluation in the two corpora of naturally-occurring dialogs recorded in real-life call center environments. These recordings are spontaneous speech. The first corpus of dialogs contains real agent-client recordings obtained from a Mobile Customer Service Center (MCSC). These recordings were made from five agents (three female, two male) and 105 clients (48 female, 57 male). This corpus also contains 121 agent-client dialogs of around 3.5 h. The second dialog corps contains agent-client recordings obtained from a Hospital Emergency Call Center (HECC). These recordings made from five agents (three female, two male) and 105 clients (48 female, 57 male) and contain 68 agent-client dialogs of around 1.8 h. Table 2 shows the proportion of turns for each emotion label for the two mixtures of emotions. These dialogs mainly cover a range of five emotions: Anger, Fear, Neutral, Sadness and Happiness. The Neural emotion almost covers the whole sentence.

### 3.3. Feature Sets

#### 3.3.1. MFCC Features

The Mel-frequency cepstral coefficients (MFCCs) are widely used in the speech analysis field. Here, the first 13 MFCCs (including the zero-order coefficient) are extracted from 25 ms. Hamming-windowed frame every 10 ms with the pre-emphasis coefficient 0.97. The mean, standard deviation, skewness, and kurtosis of these 13 MFCCs, their deltas, and double-deltas are computed as 156 features per utterance.

#### 3.3.2. Prosodic Features

The statistics of the prosodic features used in this study are similar to those used by other researchers [5,36]. However, not to form a huge feature set with 1000∼4000 parameters, a reasonably small-sized feature set is constructed. As a result, some features are omitted or replaced. For example, the mean of the positive and the negative dF0 are calculated separately to represent the upward and the downward trend, respectively, instead of the mean of all dF0. As for the energy, the minimum value of energy must be close to zero such that the min value, relative position of min, and range would not provide crucial information and hence are dropped from our feature list. The 30 prosodic features are extracted and description of this feature set is given in Table 3.

#### 3.3.3. The LLD Features

The acoustic features were those adopted in the INTERSPEECH 2009 emotion challenge [37]. This default feature set provides baseline results for both HMM and linear kernel SVM recognizers in the 2009 challenge and is totally transparent with the accessible open source openSMILE feature extraction toolkit [38]. It includes the most common features in pertaining to prosody, spectral shape, voice quality, as well as their derivatives. In details, the 16 low-level descriptors (LLD) chosen are: zero-crossing-rate (ZCR) from the time signal, root mean square (RMS) frame energy, pitch frequency (normalized to 500 Hz), harmonics-to-noise ratio (HNR) by autocorrelation function, and Mel-frequency cepstral coefficients (MFCC) 1–12 in full accordance to HTK-based computation. To each of these 16 features, the delta coefficients are included as well. Next, as depicted in Table 5, the 12 functionals: mean; standard deviation; kurtosis; skewness; minimum and maximum value, relative position, and range; and two linear regression coefficients with their mean square error (MSE); are derived for each low-level and its delta feature on a chunk basis. Thus, the final feature contains 16 × 2 × 12 = 384 attributes and is presented in Table 4.

## 4. Experiments and Results

To evaluate the efficiency of the proposed methodologies, the experiments were conducted and are described in this section. The average percentage of classification accuracy (APCA) was calculated and defined as follows:

(29) $$\text{APCA}=\left(\sum_{i=1}^{N_{\text{tot}}}(N_{\text{correct}}/N_{\text{input}})/N_{\text{tot}}\right)\times 100\%$$

where *N_correct_* is the number of test inputs correctly identified during the *i*th trial, *N_input_* is the total number of test inputs, and *N_tot_* is the number of total trials.

### 4.1. The Emotional Database

The number of instances is given in Table 5 for the four-class (Happiness, Fear, Sadness and Anger) task. In order to further evaluate the performance of the cross-corpus, the row labeled as “Mixed” is used to represent a mix of three corpora. The total of the mixed corpora is 1584 sentences. The entire data was spilt into 75% of the data representing the training set and 25% of the data representing the testing set for three databases (EMO-DB, eNTERFACE and KHUSC-EmoDB). The modeling (training) and classification (testing) process was repeated 12 times, each time with different randomly training and testing data sets.

In Table 5, we find that each emotional category has different amounts at each speech database. To be fair to the various emotional recognition rates, our experiments use a minimum number of categories from various emotional speech corpuses as a test standard. The training set and test set are not overlapped to achieve an open test. For example, 62-sentences is the minimum among the four kinds of emotions. We use 16-sentences (62 × 25%) as the number for each emotion test on the EMO-DB speech database. Then, the number of the test set and training set are 16-sentences and 46-sentences, respectively. In addition, 207-sentences is the minimum for the “Happiness” emotion for the eNTERFACE speech database. We use 52-sentences as test set and 155-sentences as training set, respectively. Because the number for each emotional category for the KHUSC-EmoDB is the same (102-sentences), the test set and training set are 26-sentences and 76-sentences, respectively.

### 4.2. The Evaluations Using Segmentation with/without Acoustic Activity Detection

To evaluate the role of AAD for recognizing emotional states, we combine the BS-Entropy-based AAD with the MFCCs and SVM classifier. The results, summarized in Table 6, show a noticeable increase for the average percentage of classification accuracy (APCA). This clearly indicates that the segment with AAD can significantly improve the accuracy for segments with silence. We can find that the segment with AAD under EMO-DB can obtain highest accuracy rate among all databases.

### 4.3. The Evaluation Results of MRTII Features with/without Contrast Cubic Curve

In this subsection, the comparison between without/with cubic curve will be then evaluated. The 2‐D spectrogram topography of the original image may contain many non-voiced parts of the information in the pronunciation. After contrast adjustment with the cubic curve, we can efficiently enhance the non-voiced pronunciation in the emotional spectrogram image, so the stress levels of emotions for speaker pronunciation can be presented in detail. Table 7 shows the evaluation results of MRTII-based feature extraction with/without the contrast cubic curve. Among the three databases, the APCA for mixed database using MRTII features with cubic curve is 84.58% against 76.82% for the evaluation without cubic curve. Based on the above experiments, we can understand that the contrast adjustment with cubic curve is helpful for the SER system. Therefore, the next experiments will use the method of contrast adjustment with cubic curve to perform the evaluations.

### 4.4. Classification Comparison

We need to select a classifier that can properly model the data and achieve better classification accuracy in order to classify the extracted features into different human emotions. A comparison of popular classifications used in emotion recognition will help us gain insight into the problem and select an appropriate method to build upon since we do not have any prior knowledge about the characteristics of the features. Consequently, we compare the performance of the Linear Discriminate Analysis (LDA), k-nearest Neighbors (KNN) and support vector machine (SVM) in this subsection. Table 8 shows the experimental results of APCA applying three classifiers with four features: MFCC, Prosodic, LLD and MRTII on the mixed database. From the comparison among the three classifiers, the evaluation results (69.23% with MFCC and 86.23% with MRTII) of the SVM classifier are higher than the results of the KNN and LDA classifiers because SVM can provide a good decision module.

### 4.5. The Feature Comparisons with MFCC, Prosodic and MRTII

#### 4.5.1. Evaluation in Artificial Databases

In this subsection, classification tests will be performed on the four databases: EMO-DB, eNTERFACE, KHUSC-EmoDB and Mixed databases. The classes also represent four different emotions. Table 9 shows the confusion between different emotions, whereas Tables 6 and 7 show the average percentage of identification accuracy. As for the Tables 7 and 8, the cubic curve and SVM classifier give the highest classification accuracy. Therefore, the confusion data in Table 9 shows only results for the SVM classifier and the cubic curve in the case of the MRTII feature.

For the comparison between the three corpora, it shows that the evaluation results of EMO-DB are higher than the results of eNTERFACE and KHUSC-EmoDB. In the EMO-DB database, the maximum APCA is achieved with the MRTII features for the “Anger” and “Happiness” emotions. LLD and Prosodic features show better results for the “Sadness” one. Similar results are observed in the eNTERFACE and KHUSC-EmoDB databases, where the MRTII features show the best results. For the eNTERFACE database, the “Sadness” emotion has maximum accuracy with the MRTII.

**Figure 4.**
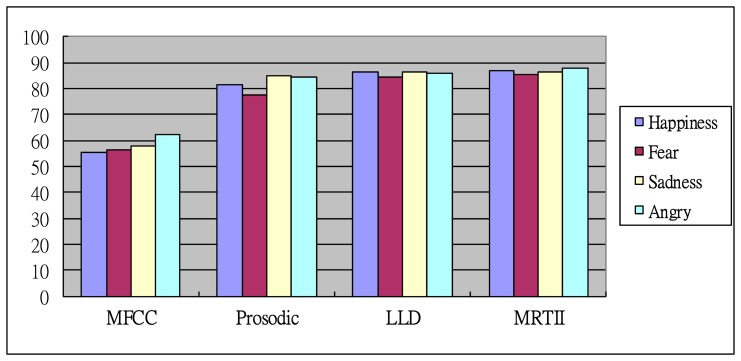
Comparison of the recognition recognitions using different feature extractions.

For the mixed database, the accuracy rate of the “Anger” emotion with MRTII is better (86.19%) than for other emotion states. In contrast, the accuracy rate of the “Happiness” emotion with the MFCC features is the lowest (48.84%) among all test data. It is found that the proposed MRTII feature can provide high accuracy when SER performs cross-corpora. In the average results of emotional classification among the four databases, the MRTII features outperformed the MFCC, Prosodic and LLD features, while the overall performance of the MFCC features was worse than for Prosodic, LLD and MRTII. The APCA achieved good accuracy (86.64%) for the MRTII features when compared to the average accuracy ranging from 58.01% to 85.80% provided by the MFCC, Prosodic and LLD features, respectively. This is due to the fact that different emotions can distribute different levels of frequency. A possible explanation is that the MRTII can easily describe the distributed frequency channel by TSWT (as shown in Table 1) to discriminate the differences between each emotion while comparing with other features. Since there are no distinct salient words used for the “Sadness” and “Fear” emotional states, the recognition performance is lower than for other emotional states. Conversely, the sentences with “Anger” emotion are often comprised of intense words. Therefore, it can achieve the best performance. In addition, the recognition recognitions using different feature extractions are also summarized in Figure 4. The results demonstrate that the proposed MRTII-based feature extraction combined with BS-Entropy-based AAD can achieve better recognition accuracy than others.

#### 4.5.2. Evaluation in Real-life Corpora

Table 10 shows the evaluation of blended emotional recognition in real-life corpora from 121 agent-client dialogs in MCSC and 68 agent-client dialogs in HECC. Compared to Table 9, the performance in Table 10 is obviously degraded in real-life recordings due to the fact that the considered emotions of different intensity are blended into dialogs. Consequently, the best rate obtained is only 73.68% for the proposed MRTII feature and SVM. For the existing studies, the accuracy of 73.68% is enough to be considered real-life emotional recognition. Based on the findings from Table 10, we can know the proposed MRTII-based emotional algorithm still performs well for spontaneous speech compared to the other features. It is well-known that spontaneous speech blends various emotions into a sentence. Based on the findings, an AAD algorithm is more critical for high accuracy of emotional discrimination. Through the BS-Entropy-based AAD method, the correct emotional VAS can be first extracted from spontaneous speech in spite of change in amplitude of emotional input or in background noise-level. We can summarize that the BS-Entropy-based AAD can make the proposed MRTII-based algorithm perform well whether in an artificial corpus or in spontaneous speech, especially in a real-life condition.

## 5. Conclusions

In this paper, a novel feature set for emotion classification in speech is proposed. The system makes use of MRTII for feature representation and a SVM as the recognizer. The proposed MRTII-based feature extraction algorithm including BS-Entropy based acoustic activity detection (AAD) and the multi-resolution texture analysis. The BS-Entropy-based AAD method is first utilized to determine the voice-active segments (VAS). We find that the calculation of the spectral entropy parameter implies that the spectral entropy depends only on the variation of the spectral energy but not on the amount of spectral energy. In real-life environment, the spectral entropy parameter is robust against changing signal levels, even though signal amplitude varies with the emotional state. So, the utilized BS-Entropy-based AAD is benefit for real-life emotional recognition in speech. In order to enhance image contrast, cubic curve compensation is then used. In addition, the tree-structured 2-D wavelet packet transform (TSWT) can be used to generate the desired multi-resolution spectrogram images. Then, the multi-resolution texture image information can be successfully extracted by Laws' texture energy measures from the desired subimages. Next, we also evaluate different classifiers with various feature sets for the classification of emotional speech. We observed that the SVM classifier with the MRTII features is the best choice among the three tested classifiers: SVM, KNN and LDA. In three artificial corpora: EMO-DB, eNTERFACE and KHUSC-EmoDB and a mixed database, the results of the experiments show that an average accuracy of 86.64% and best accuracy of 91.32% can be achieved in classifying the five basic emotions individually. In real-life corpora, the accuracy of 73.68% is enough to be considered real-life emotional recognition compared to other features.

In summary, we find that through the BS-Entropy-based AAD method, the correct emotional VAS can be first extracted from spontaneous speech in spite of change in amplitude of emotional input or in background noise-level. In addition, the MRTII feature set derived from time-frequency representation can perform well for emotion classification. It is critical to extract features that capture the major temporal-spectral characteristics of signals to achieve a high accuracy in speech emotional classification, especially in real-life condition.

Future work on the classification of emotional speech could combine with visual emotion recognition from facial features for real-life emotional discrimination. This paper will apply the concept of texture image information to human emotional states from audiovisual signals. Therefore, the audiovisual signals, including speech and image, can be both processed with image processing to build a one-kernel two-module (OKTM) system. Based on the OKTM system, we anticipate that the costs for SER system will be significantly cut. In addition, we find that different languages may cause variable performance in emotion recognition. This is worth exploring as another future SER research direction.

## Figures and Tables

**Table 1.** The first 4 dominant channels for five types of emotion.

**Emotion**	**Dominant Frequency Channel**			

	**First Channel**	**Second Channel**	**Third Channel**	**Fourth Channel**
**Fear**	LL1, LL2, HL3, LH4	LL1, LL2, HH3, LL4	LL1, HL2, HL3, HH4	LL1, HL2, HH3, HH4
**Neutral**	LL1, LL2, LL3, LL4	LL1, LL2, LL3, LH4	LL1, LL2, LH3, LL4	LL1, LL2, HH3, HL4
**Sadness**	LL1, LL2, HL3, LH4	LL1, LL2, HH3, LH4	LL1, HL2, HH3, HL4	LL1, HL2, HL3, HH4
**Anger**	LL1, HL2, LH3, LL4	LL1, HL2, LH3, LH4	LH1, HL2, LL3, LL4	LH1, HL2, LL3, LH4
**Happiness**	LL1, HL2, LH3, LL4	LL1, HL2, LH3, LL4	LL1, HL2, HH3, HL4	LH1, HL2, HL3, HH4

**Table 2.** The proportion of each emotion label in the dialog Corpus 1 and Corpus 2.

**Corpora**		**Anger (%)**	**Fear (%)**	**Sadness (%)**	**Happiness**	**Others**	**Neutral (%)**
**121 agent-client**	**Client**	5.7%	1.5%	2.4%	3.5%	3.05%	83.85%
**dialogs in MCSC**	**Agent**	1.2%	0.4%	0.3%	5.2%	2.74%	94.16%

**68 agent-client**	**Client**	9.23%	5.8%	6.8%	0.3%	1.64%	76.23%
**dialogs in HECC**	**Agent**	1.8%	1.0%	1.2%	2.6%	1.54%	91.86%

**Table 3.** Prosodic features set.

F0 (8 features)	mean, std, max value, relative position of max, min value, relative position of min, range, number of local max point
dF0 (8 features)	mean of positive, mean of negative, std, max value, relative positive of max, min value, relative position of min, ratio of positive
logE (3 features)	std, max value, relative position of max
dlogE (8 features)	mean of positive, mean of negative, std, max value, relative position of max, min value, relative position of min, ratio of positive
Duration (3 features)	speaking rate, std of voiced duration, mean pause time

**Table 4.** The LLD Features used in INTERSPEECH 2009 emotion challenge [37].

LLD(16 × 2)	Functionals (12)
(delta) ZCR	Mean
(delta) RMS	Energy standard deviation
(delta) F0	Kurtosis, skewness
(delta) HNR	Extremes: value, rel. position, range
(delta) MFCC 1–12	Linear regression: offset, slope, MSE

**Table 5.** Description of the collected speech database.

**Emotional Class**	**Happiness**	**Fear**	**Sadness**	**Anger**	***Total***

**Corpora**					
**EMO-DB**	71	69	62	127	*329*
**eNTERFACE**	207	215	210	215	*847*
**KHUSC-EmoDB**	102	102	102	102	*408*

***Mixed***	*380*	*386*	*374*	*444*	*1584*

**Table 6.** The average percentage of classification accuracy (APCA) of performance comparisons with/without AAD.

**Database**	**Segment without AAD**	**Segment with AAD**
EMO-DB	66.54%	69.23%
eNTERFACE	60.21%	64.58%
KHUSC-EmoDB	59.58%	62.36%

**Table 7.** The APCA for the Mixed database using MRTII with/without cubic curve.

**Database**	**MRTII without Cubic Curve**	**MRTII with Cubic Curve**
EMO-DB	78.42%	84.53%
eNTERFACE	77.58%	81.34%
KHUSC-EmoDB	76.24%	80.15%
Mixed	76.82%	84.58%

**Table 8.** The APCA for the Mixed database using MFCC and MRTII features combined with SVM, KNN and LDA classifiers.

**Classifier**	**MFCC**	**MRTII**
SVM [39]	69.23%	86.23%
KNN	64.58%	84.76%
LDA [40]	61.14%	83.85%

**Table 9.** Confusion table for Artificial Databases using MFCC, Prosodic, LLD and MRTII.

**Database**	**MFCC**				**Prosodic**				**LLD**				**MRTII**			

	**Ha**	**Fe**	**Sa**	**An**	**Ha**	**Fe**	**Sa**	**An**	**Ha**	**Fe**	**Sa**	**An**	**Ha**	**Fe**	**Sa**	**An**
**EMO-DB**	63.59	60.25	57.28	66.48	85.37	82.56	89.39	86.49	90.12	88.72	90.65	89.37	90.54	89.28	88.43	91.32
**eNTERFACE**	57.94	59.31	64.52	60.28	82.49	79.38	87.26	85.21	87.29	85.83	88.39	86.58	88.28	84.67	89.48	87.92
**KHUSC-EmoDB**	50.48	56.29	55.38	61.28	80.27	75.38	82.39	83.67	84.22	82.95	85.93	85.27	84.28	84.48	83.02	86.58
***Mixed***	*48.84*	*50.49*	*54.91*	*60.84*	*78.82*	*72.28*	*80.39*	*81.97*	*83.83*	*79.69*	*81.28*	*82.73*	*84.88*	*82.91*	*83.95*	*86.19*

**Average**	58.01%				82.08%				85.80%				86.64%			

Ha: Happiness; Fe: Fear; Sa: Sadness; An: Angry.

**Table 10.** Confusion table for real-life corpora using MFCC, Prosodic, LLD and MRTII.

**Database**	**MFCC**				**Prosodic**				**LLD**				**MRTII**			
Emotional Type	Ha	Fe	Sa	An	Ha	Fe	Sa	An	Ha	Fe	Sa	An	Ha	Fe	Sa	An
121 agent-client dialogs in MCSC	42.8	58.3	57.3	58.7	64.8	65.3	66.8	65.7	69.5	70.1	72.6	70.3	75.4	74.4	74.8	76.5
68 agent-client dialogs in HECC	43.6	54.7	53.6	54.2	60.3	62.6	65.5	66.8	67.9	68.4	69.2	70.9	71.5	72.6	71.7	72.5

Average (%)	52.90%				64.73%				69.86%				73.68%			

Ha: Happiness; Fe: Fear; Sa: Sadness; An: Angry.
